# A Multicenter Study into Burnout, Perceived Stress, Job Satisfaction, Coping Strategies, and General Health among Emergency Department Nursing Staff

**DOI:** 10.3390/jcm9041007

**Published:** 2020-04-02

**Authors:** Silvia Portero de la Cruz, Jesús Cebrino, Javier Herruzo, Manuel Vaquero-Abellán

**Affiliations:** 1 Department of Nursing, Pharmacology and Physiotherapy, Faculty of Medicine and Nursing, University of Córdoba, Avda. Menéndez Pidal, S/N, 14071 Córdoba, Spain; mvaquero@uco.es; 2 Department of Preventive Medicine and Public Health, Faculty of Medicine, University of Seville, Avda. Doctor Fedriani, S/N, 41009 Seville, Spain; jcebrino@us.es; 3 Department of Psychology, Faculty of Education, University of Cordoba, C/ San Alberto Magno, S/N, 14071 Córdoba, Spain; ed1hecaf@uco.es

**Keywords:** adaptation, psychological, burnout, emergency service hospital, job satisfaction, nursing staff, occupational stress

## Abstract

Burnout is a major problem among nurses working in emergency departments and is closely related to a high turnover of personnel, nursing errors, and patient dissatisfaction. The aims of this study were to estimate burnout, perceived stress, job satisfaction, coping and general health levels experienced by nurses working in emergency departments in Spain and to analyze the relationships between sociodemographic, occupational, and psychological variables and the occurrence of burnout syndrome among these professionals. A cross-sectional study was conducted in four emergency departments in Andalusia (Spain) from March to December 2016. The study sample was composed of *n* = 171 nurses. An ad hoc questionnaire was prepared to collect sociodemographic and work data, and the Maslach Burnout Inventory, the Perceived Stress Scale, the Font–Roja Questionnaire, the Brief Cope Orientation to Problem Experience and the General Health Questionnaire were used. The prevalence of high burnout was 8.19%. The levels of perceived stress and job satisfaction were moderate. The most frequent clinical manifestations were social dysfunction and somatic symptoms, and problem-focused coping was the strategy most used by nurses. Lack of physical exercise, gender, years worked at an emergency department, anxiety, social dysfunction, and avoidance coping were significant predictors of the dimensions of burnout.

## 1. Introduction

Burnout is a psychological syndrome emerging as a prolonged response to chronic interpersonal stressors at work. The three key dimensions of this response are: (i) emotional exhaustion (EE), or a loss of enthusiasm in one’s work; (ii) depersonalization (DP), or an impersonal response to patients; and (iii) personal accomplishment (PA), or a feeling of carrying out one’s job successfully [1,2]. Burnout has been included in the 11^th^ Revision of the International Classification of Diseases as an occupational phenomenon [3] affecting a broad spectrum of workers [4]. Healthcare professionals are more likely to develop burnout [5], and nurses in particular are among the major risk groups [6]. Studies have reported high values in this respect for nurses [7,8].

However, the various clinical contexts differently affect the nurses’ susceptibility to burnout [9,10,11]. It is estimated that 26% of nursing professionals working in emergency departments (EDs) suffer from burnout, defined as a state of depletion of resources of an employee as a result of negative perception of the work environment, and is characterized by EE, DP, and lack of PA [12]. This prevalence is higher than in other medical specialties [13,14].

Emergency departments (EDs) are experiencing increasing service demands [15,16], which can lead to increased workload and pressures on ED nursing personnel [17]. Moreover, in ED, waiting times, a demanding public, overcrowding, and inadequate human resources are common stressors [17,18]. Similarly, occupational stress, defined as the perception of a discrepancy between environmental demands (stressors) and individual capacities to fill these demands determined by work organization, work design, and labor relations [19], is also associated with burnout [20]. The prevalence of job stress is increasing globally [21]. Over a number of studies, between 27% to 46.9% of ED nursing staff reported a high level of occupational stress [22,23]. This situation may, in turn, lead to nursing errors and lower patient satisfaction [24].

Equally as troubling is job dissatisfaction. High levels of stress and burnout are linked to lower satisfaction in nursing professionals [25]. Job satisfaction is considered a global problem due to the potential impact on the safety of patients and the quality of working life of nursing staff [26]. Job satisfaction is defined as “the pleasure or a positive emotional state resulting from the appraisal of one’s job or job experiences” [27]. It is estimated that over 50% of ED nursing personnel are dissatisfied with their work [28]. Job dissatisfaction may lead to undesired workplace outcomes, such as increased turnover intention and nurses’ absenteeism [29].

Although occupational stress and burnout are common among ED nursing personnel, not all ED nursing staff show altered levels of the aforementioned dimensions. Some professionals will thrive in the same seemingly stressful environment. One important factor that may influence the likelihood of job stress and burnout is an individual’s coping style, defined as the cognitive and behavioral efforts applied by an individual to manage stress [30]. Researchers [18,31] have found a significant relationship between adaptive coping strategies—understood as those responses that actively and directly deal with a stressor, improving the adaptation outcome [32]—and a decreased level of burnout. In ED, staff are more likely to use maladaptive coping strategies—defined as negative and ineffective cognitive or behavioral responses to stress and anxiety [33]—compared with other clinical areas [34].

Despite occupational stress, job satisfaction, and coping styles are associated with general health of nurses, burnout is most significantly associated with general health [35], resulting in poor physical and mental health manifestations, such as headaches, depression, and insomnia [25,35]. In Spain, the economic cost of mental and behavioral disorders attributable to work is estimated at between €150 and €372 million, which represents 0.24% to 0.58% of the total annual health expenditure [36].

To the best of our knowledge, this study is the first research in ED nurses that measures and compares these different types of psychological states at the same time. It is important to identify the most significant relationships between occupational stress, burnout, coping, job satisfaction, and general health so that evidence-based policy and practice aimed at promoting healthy working environments for nurses can be promptly developed. The main objectives of this study were therefore as follows: to estimate the burnout, perceived stress, job satisfaction, coping and general health levels experienced by nurses working in Spanish EDs and to analyze the relationship between the sociodemographic, occupational, and psychological variables and the occurrence of burnout syndrome among these professionals.

## 2. Materials and Methods

### 2.1. Study Design

A quantitative, observational, cross-sectional, multicenter study.

### 2.2. Study Settings

The study was carried out at the EDs of four hospitals in Andalusia (southern Spain). ED 1 has 165,520 visits per year and a reference population of 481,296 inhabitants; ED 2 has 135,000 visits per year and a reference population of 461,078 inhabitants; ED 3 has 48,000 visits per year and a reference population of 62,234 inhabitants; ED 4 has 18,000 visits per year and a reference population of 24,287 inhabitants.

### 2.3. Participants and Sample

In order to assess the suitability of the study population, the required sample size was calculated using Epidat version 3.1 (General Directorate of Public Health, Galicia, Spain). We considered a 95% confidence level, an absolute precision of 3%, and a prevalence of burnout among ED nursing staff of 26% [12]. With these data, the estimated minimum sample size was 169 subjects.

212 cover letters with the questionnaires were sent in sealed envelopes to all the nurses in the four EDs selected. This number comprised the total number of nurses employed by the four EDs at the start of the study which complied with the inclusion criteria, which included all the active nurses during data collection who had worked at the ED for at least one year. The exclusion criteria were nurses on sick leave or unpaid leave during data collection. In the end, 171 questionnaires were completed (80.66% response rate).

### 2.4. Data Collection

The data were collected from March to December 2016. The study data were compiled for the following sociodemographic, occupational, and psychological variables: sociodemographic variables, including sex (male, female), age (years), marital status (single, married, separated/divorced, widowed), daily physical exercise (yes, no), and daily tobacco use (yes, no). The occupational variables included type of employment contract (permanent, indefinite, part-time), time of service at the ED (years), and work experience (years). The psychological variables were burnout, perceived stress, job satisfaction, coping strategies, and general health.

Burnout syndrome was measured using the Maslach Burnout Syndrome (MBI) [37] adapted for the Spanish population [38]. This instrument contains 22 items scored on a seven-point Likert response scale ranging from 0 (never) to 6 (every day). The MBI result is presented with reference to three dimensions: EE (nine items), DP (five items), and PA (eight items). The dimensions were categorized into low, average, and high levels considering the cut-off points established previously in the literature [39,40]: EE: low: 0–18, medium 19–26, high: ≥ 27; DP: low: 0–5, moderate: 6–9, high: ≥ 10; and PA: low: 0–33, moderate: 34–39, high: ≥ 40. Low scores for EE and DP and high ones for PA indicate the absence of burnout. The rest of the cases are indicative of burnout (high level of burnout was defined by high scores for EE and DP and low ones for PA, and moderate level of burnout was determined by the rest of the cases). The following reliability coefficients (α) for the MBI scales were calculated: EE (α = 0.90), DP (α = 0.60), and PA (α = 0.81).

Perceived stress was measured using the Perceived Stress Scale [41] adapted for the Spanish population [42]. The main characteristic of perceived stress is that the response of an individual is not based exclusively on the characteristics of the stimulus, but is greatly influenced by personal and contextual factors [42]. This tool evaluates the degree to which individuals believe their life has been unpredictable, uncontrollable, and overloaded over the previous month. The assessed items are general in nature rather than focusing on specific events or experiences, and it contains 14 items scored on a five-point Likert response scale ranging from 0 (never) to 4 (very often). The overall perceived stress is obtained by adding the scores of the 14 items. The results range from 0 to 56 points. Perceived stress increases with higher scores. A score between 20–22 points is considered a level of perceived stress within the normal range [42]. The internal consistency value measured in terms of the Cronbach’s alpha for the Perceived Stress Scale was 0.73.

The questionnaire used to assess job satisfaction was the Font–Roja questionnaire [43]. It consists of 24 items and explores 9 dimensions that determine a professional’s level of satisfaction: job satisfaction, work-related tension, professional competence, job pressure, professional promotion, interpersonal relationship with their superiors, interpersonal relationship with coworkers, extrinsic characteristics of status, and job monotony. Each item is valued using a Likert scale, with values ranging from 1 (totally disagree) to 5 (totally agree). The overall job satisfaction is obtained by the addition of the scores of the 24 responses and ranges from 24 to 120 points. The higher the score, the greater the job satisfaction. In this study, the Cronbach’s alpha coefficient was 0.83 for the overall job satisfaction.

The Spanish version [44] of the Brief COPE [45] was used to evaluate how individuals cope with stressful situations. Coping is defined as “constantly changing cognitive and behavioral efforts to manage specific external and/or internal demands that are appraised as taxing or exceeding the resources of the person” [30]. This questionnaire is made up of 28 items on a four-point Likert scale from 0 (I absolutely never do this) to 3 (I do this often). The items are grouped into 14 subscales measuring 3 strategies: problem-focused coping (active coping, planning, and search for instrumental support), emotion-focused coping (search for emotional support, positive reinterpretation, negation, acceptance, religion, and humor), and avoidance coping (self-distraction, venting, behavior disconnection, substance use, self-blame). A higher score indicates a higher use of the coping strategy. Cronbach’s alpha coefficients for the 3 coping strategies were: α = 0.83 (problem-focused coping), α = 0.85 (emotion-focused coping), and α = 0.90 (avoidance coping).

Health status was measured using the General Health Questionnaire (GHQ) [46] validated for the Spanish population [47]. This tool contains 28 items in 4 subscales referring to somatic symptoms (7 items), anxiety (7 items), social dysfunction (7 items), and depression (7 items). Answers follow a 4-point Likert scale, ranging from 0 (less than usual) to 3 (much more than usual). The total score for each scale ranged from 0 to 21 points. The total score of the GHQ ranged from 0 to 84 points. A higher score is related to worse health status. The following reliability coefficients (α) for the total score of the GHQ and for the scales were calculated: α = 0.88 (total GHQ), α = 0.85 (somatic symptoms), α = 0.81 (anxiety), α = 0.85 (social dysfunction), and α = 0.90 (depression).

Data research is available as Supplementary File.

### 2.5. Ethical Considerations

The study was approved by the clinical research ethics committee (approval number 249, reference 3050). A cover letter explaining the voluntary and confidential nature of the study was delivered to all ED healthcare personnel.

### 2.6. Data Analysis

A descriptive analysis was performed using the means and the standard deviations for the quantitative variables, and frequencies and percentages for the categorical variables. The Kolmogorov–Smirnov test was used to check the normality of the variables. Student’s *t-*, Mann–Whitney, Analysis of variance and Kruskal–Wallis tests were used to analyze the relationships between the sociodemographic, occupational, and psychological characteristics and the MBI dimensions. Correlations between the quantitative variables were tested using the Pearson correlation and the Spearman’s Rho tests. Three univariate linear regressions were created in order to assess the relationship between the sociodemographic, occupational, and psychological characteristics and each MBI dimension. Those variables that showed a statistically significant relationship with each of the considered dimensions (*p* < 0.05) were subsequently included in a multivariate linear regression model. In this way, 3 elimination multiple linear regression models were created for each MBI dimension (EE, DP, PA). For purposes of the multivariate analysis, the variables were reorganized as follows: marital status (married, not married) and type of employment contract (permanent, non-permanent). All the results were considered statistically significant with the *p*-value < 0.05. The statistical analyses were performed with statistical package G-Stat V.2.0 (GlaxoSmithKline S.A., Madrid, Spain).

## 3. Results

### 3.1. Characteristics of Participants

A total of 171 nurses participated in the study. The participants’ mean age was 47.85 (8.11) years, and 73.10% were women. Of the group, 60.23% were single, 48.54% did daily physical exercise, and 67.25% had a permanent contract. Other sociodemographic and work characteristics are shown in Table 1.

### 3.2. Descriptive Analysis of Burnout

**Figure 1:**
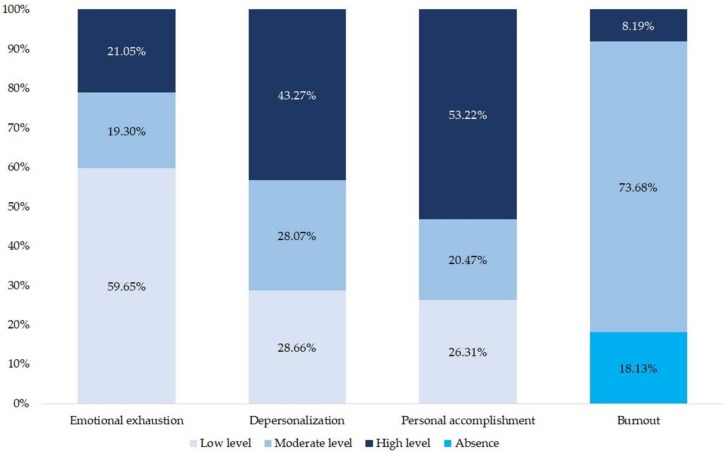
Levels of emotional exhaustion, depersonalization, personal accomplishment, and burnout.

As regards the levels of burnout (Figure 1), ED nurses had a higher prevalence of low levels of EE (59.65%) and high levels of DP (43.27%) and PA (53.22%). The prevalence of high level of burnout was 8.19%.

### 3.3. Descriptive and Correlational Analysis of Burnout, Perceived Stress, Job Satisfaction, Coping Strategies, and General Health

As shown in Table 2, the average perceived stress and job satisfaction scores among all the workers were found to be 21.30 (5.94) and 67.19 (6.98) points, respectively. Higher scores of EE were positively correlated with anxiety (*p* = 0.001) and social dysfunction (*p* = 0.002). A significant negative correlation was also found between perceived stress and job satisfaction (*p* = 0.0004). Burnout variables EE and DP and the subscales corresponding to the GHQ showed positive relationships with avoidance coping (*p* < 0.01 and *p* < 0.001, respectively). Problem-focused coping was negatively correlated with depression (*p* = 0.003) and social dysfunction (*p* = 0.0002). It should also be noted that a significant positive correlation was found between emotion-focused coping and depression (*p* = 0.002).

### 3.4. Comparative Analysis of Sociodemographic, Occupational Characteristics, and The Dimensions of Burnout

The differences between the average burnout (EE, DP, and PA) scores were evaluated according to the participants’ sociodemographic data and occupational characteristics. Accordingly, those who did not take part in daily physical exercise had higher mean DP (*p* = 0.005) scores. There were negative significant relationships between PA and age (*p* = 0.03), time of service at the ED (*p* = 0.03), and work experience (*p* = 0.02) (Table 3).

### 3.5. Multivariate Linear Regression Models

Table 4 shows the multivariate linear regression models obtained for each of the MBI dimensions. The results indicate that the use of avoidance coping (*p* = 0.03), anxiety (*p* = 0.02), social dysfunction (*p* = 0.02), and being female (*p* = 0.01) were statistically significant predictors of EE. DP was determined by the absence of daily physical exercise (*p* = 0.006), being female (*p* = 0.01), and the use of avoidance coping (*p* = 0.03). PA seems to be influenced by the years worked at EDs (*p* = 0.03).

## 4. Discussion

In this study, 21.05% and 43.27% of the participants had high levels of EE and DP, respectively. 26.31% had low PA. Among the nursing staff, the prevalence of each of the 3 dimensions of burnout according to the MBI range was as follows: high level of EE (20–44%), high level of DP (23–51%), and low level of PA (15–44%) [11]. Regarding the prevalence of burnout, the study carried out in ED nurses showed that 3.40% suffered from high levels of burnout [48]. This prevalence is more than half than that obtained in our study.

The participants’ perceived stress score was within the normal range, similar to the results of Mirhagi and Sarabien [49]. However, lower scores have been reported in the literature. Hutchinson et al. [50] found that the average score of perceived stress among ED medical personnel was 15.53 points, and Wong et al. [51] reported 12.30 points. These variations may be due to the use of the 10-item version of the Perceived Stress Scale. Although the perceived stress score obtained was not high, we consider that the level of perceived stress is in fact higher among ED nursing professionals due to lack of personnel, work overload, shift work, role ambiguity, lack of autonomy, rapid technological changes, and increased pressure in decision-making [52,53].

Despite the fact that the impact of working in an ED on the level of stress and burnout among nurses has now been established [54,55,56], less is known about its impact on job satisfaction. In the present study, it was found that the level of job satisfaction among the participants was moderate, which is consistent with another study [28]. In the field of health, most of the studies present similar results: medium-high level of job satisfaction in medical staff [57] and lower levels among nursing personnel [58,59]. In EDs, nurses show a higher degree of dissatisfaction than nurses working in other specialties, due mainly to understaffing and poor professional status [60]. In addition, we found a negative correlation between job satisfaction and perceived stress, which matched results from other studies [61,62].

In the current study, somatic symptoms and social dysfunction were the most frequent clinical manifestations among ED nurses. This is consistent with the results of another study carried out among emergency and intensive nursing staff [63]. The use of adaptive coping styles produces a positive effect on physical and psychological well-being, management of stress, and overall performance among healthcare professionals [64], which is related to an improvement in the quality of care, greater patient safety, and a fall in health service costs [65]. This is congruent with our results that showed that the use of problem-focused coping reduced both social dysfunction and anxiety and depressive symptomatology. We found that the most commonly used coping strategy was problem-focused coping and the least common was avoidance coping, as in similar studies [18,64,66,67,68]. The use of avoidance coping may be explained by the low level of personal suffering due to the high turnover rate occurring in EDs [56]. In the multivariate analysis, EE and DP were determined positively by the use of avoidance coping, which was consistent with the findings of other authors [69,70]. It should be noted that some authors have long argued that the DP dimension is in fact a coping style [71,72]. In addition, this type of coping was positively related to somatic symptoms, anxiety, social dysfunction, and depression. These results were similar to those of Yates et al. [73]. Nevertheless, avoidance coping may be the best option for ED personnel when an event occurs in order to avoid emotional involvement [74].

We found that EE and DP were influenced positively by being a female. However, this result should be viewed with caution due to the sample of our study consisting predominantly of women. While previous studies noted that gender is an important variable in EE and that women experience more burnout than men [75,76], there are other studies which suggest that the burnout is not associated with gender in EDs [77,78]. The significantly higher EE scores in women may be due to the social role played by women and their effort to strike a better work-life balance [79]. In addition, work-family conflicts are considered important risk factors in the development of burnout among women [80]. Regarding the age of ED nurses, Gökçen et al. [81] determined that this was positively related to EE. On the other hand, Schooley et al. [75] also found the same relationships and a significant positive relationship with DP. Lloyd et al. [82] showed that with age, the level of DP decreased, while the level of PA increased in ED physicians. In the present study, a significant negative relationship was found between age and the PA level, which is due to the fact that, over time, daily work with people tends to lead to feelings of personal inadequacy and low professional self-esteem as a result of the lack of concern for the problems of others and the loss of empathy [81].

In this study, no relationship was found between marital status and the dimensions of burnout. In this, the findings from the literature are again unclear and contradictory. Some authors suggest that burnout is associated with people who have no partner [83], while others argue otherwise [78] and find no relationship between these variables [75]. These disagreements highlight the importance of exploring the role of marital status in the workplace.

The role of lifestyles in ED healthcare professionals’ burnout levels needs to be studied extensively [79]. Furthermore, no relationship was found between smoking and the dimensions of burnout. This result is similar to that obtained in ED physicians [84]. DP, in the multivariate analysis, was determined by the absence of daily physical exercise. Likewise, Goldberg et al. [85] reported that low levels of physical exercise were a predictor of burnout in ED personnel. It has been suggested that regular physical exercise facilitates psychological detachment from work and increases people’s self-efficacy. As a result, ED nurses may feel more able to cope with their work duties and may find the tasks less demanding, which reduces the risk of burnout. In addition, regular physical exercise may result in the body recovering faster after exposure to stress and may induce changes in several neurotransmitters and neuromodulators, leading to a better mood and increased energy, thus reducing the risk of burnout [86].

As regards job characteristics, the correlational analysis showed an inverse relationship between work experience and PA. In the multivariate analysis, PA was influenced negatively by the years worked in EDs. Working in an ED involves dealing with unexpected situations, patients who have life-threatening pathologies, and more frequent attacks or assaults than other specialized medical units, which may produce lack of assertive skills in nursing professionals and, as a result, low PA [11,87]. However, here, too, there are conflicting findings. While Popa et al. [88] found no relationship between years worked in EDs and the level of burnout, other studies have found a significant positive relationship evident in two periods, corresponding to workers in the first two years of their professional career and those with over ten years of experience. In these stages, the relationship with burnout is lower [85,89].

No differences were found between the type of employment contract and the dimensions of burnout, which was not consistent with the results from Garcia et al. [90], who revealed that ED staff with permanent contracts had a lower level of DP than those with part-time contracts.

Our study has certain limitations. First of all, because of the cross-sectional study design, it is not possible to establish any cause-effect relationships. Secondly, findings may not necessarily be representative, as a convenience sample was used. Thirdly, the study was carried out only in the region of Andalusia, which may limit the generalization of the results. In further studies, it would be interesting to consider using a wider geographical range, and to use longitudinal research methods and randomized sampling.

It is vital for health services to be aware of the relationships between burnout, perceived stress, job satisfaction, coping strategies, and general health. Since ED nursing professionals provide a valuable service to the community, the levels of these factors should be taken into account, as they have an important impact on patients, as well as on the general population. Understanding the influence that work characteristics have on burnout is crucial to inform policy and practice in designing suitable interventions to prevent illnesses and improve motivation among ED nurses.

## 5. Conclusions

High burnout affects 8.19% nurses working in the EDs of four hospitals in the region of Andalusia. Perceived stress is within the normal range and job satisfaction level is moderate. Problem-focused coping is the most commonly used strategy, and somatic symptoms and social dysfunction are the most frequently experienced clinical manifestations. The absence of physical exercise, gender, years worked in EDs, anxiety, social dysfunction, and avoidance coping are the main predictors of burnout.

## Figures and Tables

**Table 1:** Sociodemographic and occupational characteristics of the participants.

Variables	*N* = 171 *n* (%)
Sex	
Male	46 (26.90)
Female	125 (73.10)
Marital status	
Married	39 (22.81)
Single	103 (60.23)
Separated/divorced	22 (12.87)
Widowed	7 (4.09)
Daily physical exercise	
Yes	83 (48.54)
No	88 (51.46)
Daily tobacco use	
Yes	86 (50.29)
No	85 (49.71)
Type of employment contract	
Permanent	115 (67.25)
Indefinite	33 (19.30)
Part-time	23 (13.45)
**Variables**	**Mean (Standard Deviation)**
Age (years)	47.85 (8.11)
Time of service at the emergency department (years)	12.76 (9.77)
Work experience (years)	22.83 (8.54)

**Table 2:** Relationship between burnout, perceived stress, job satisfaction, coping strategies, and general health.

	M (SD)	EE	DP	PA	A	B	C	D	GHQ	PSS	FRQ	PFC	EFC	AC
		CC (*p*-Value)	CC (*p*-Value)	CC (*p*-Value)	CC (*p*-Value)	CC (*p*-Value)	CC (*p*-Value)	CC (*p*-Value)	CC (*p*-Value)	CC (*p*-Value)	CC (*p*-Value)	CC (*p*-Value)	CC (*p*-Value)	CC
EE	17.04 (11.25)	1												
DP	8.47 (6.34)	0.62 (0.0001)	1											
PA	37.85 (8.39)	−0.39 (0.0001)	−0.38 (0.0001)	1										
A	7.96 (3.98)	0.15 (0.06)	0.008 (0.91)	−0.04 (0.64)	1									
B	7.88 (4.18)	0.08 (0.001)	−0.003 (0.97)	−0.06 (0.45)	0.68 (0.0001)	1								
C	8.70 (2.88)	0.14 (0.002)	−0.04 (0.57)	−0.06 (0.42)	0.50 (0.0001)	0.46 (0.0001)	1							
D	5.50 (5.27)	0.12 (0.11)	0.01 (0.86)	0.07 (0.35)	0.62 (0.0001)	0.64 (0.0001)	0.50 (0.001)	1						
GHQ	30.04 (13.57)	0.15 (0.06)	−0.008 (0.92)	−0.01 (0.86)	0.85 (0.0001)	0.85 (0.0001)	0.70 (0.0001)	0.87 (0.0001)	1					
PSS	21.30 (5.94)	0.007 (0.93)	0.13 (0.10)	−0.05 (0.54)	−0.16 (0.003)	−0.12 (0.12)	−0.01 (0.86)	−0.04 (0.64)	−0.10 (0.19)	1				
FRQ	67.19 (6.98)	−0.04 (0.57)	−0.003 (0.97)	−0.06 (0.41)	−0.07 (0.36)	−0.05 (0.56)	−0.03 (0.66)	−0.11 (0.14)	−0.07 (0.35)	−0.05 (0.0004)	1			
PFC	1.51 (0.51)	−0.10 (0.18)	−0.16 (0.07)	0.07 (0.40)	−0.16 (0.004)	−0.20 (0.0002)	−0.25 (0.0002)	−0.25 (0.003)	−0.26 (0.0005)	0.02 (0.81)	−0.07 (0.37)	1		
EFC	1.28 (0.36)	0.04 (0.62)	0.03 (0.73)	0.01 (0.91)	0.12 (0.13)	0.10 (0.18)	0.06 (0.43)	0.21 (0.002)	0.16 (0.0006)	−0.07 (0.39)	−0.10 (0.19)	0.26 (0.0004)	1	
AC	1.09 (0.45)	0.13 (0.004)	0.10 (0.0002)	0.03 (0.66)	0.44 (0.0001)	0.52 (0.0001)	0.42 (0.0001)	0.65 (0.0001)	0.63 (0.0001)	0.003 (0.97)	−0.06 (0.44)	−0.12 (0.13)	0.37 (0.0003)	1

M: Mean; SD: Standard deviation; EE: Emotional exhaustion; DP: Depersonalization; PA: Personal accomplishment; A: Somatic symptoms; B: Anxiety; C: Social dysfunction; D: Depression; GHQ: Total score of the General Health Questionnaire; PSS: Total score of the Perceived Stress Scale; FRQ: Total score of the Font–Roja Questionnaire; PFC: Problem-focused coping; EFC: Emotion-focused coping; AC: Avoidance coping; CC: Correlation coefficient.

**Table 3:** Comparison of participants’ burnout and sociodemographic and occupational characteristics.

Variables	EE (Points)	*p*-Value	DP (Points)	*p*-Value	PA (Points)	*p*-Value
	M (SD)		M (SD)		M (SD)	
Sex						
Male	15.80 (10.60)	0.02	7.69 (5.99)	0.007	38.18 (8.07)	0.40
Female	20.39 (12.35)		10.61 (6.80)		36.96 (9.22)	
Marital status						
Married	16.67 (10.25)		9.23 (5.40)		38.36 (8.37)	
Single	17.21 (11.38)		8.89 (6.32)		37.70 (8.34)	
Separated/divorced	15.91 (11.65)	0.88	6.18 (7.12)	0.13	37.32 (9.32)	0.52
Widowed	20 (15.02)		5.29 (7.67)		39 (7.57)	
Daily physical exercise						
Yes	15.22 (10.50)	0.09	7.07 (5.56)	0.005	38.33 (8.17)	0.48
No	18.74 (11.71)		9.80 (6.76)		37.41 (8.61)	
Daily tobacco use						
Yes	16.70 (10.87)	0.69	8.47 (6.15)	0.99	38.21 (8.11)	0.58
No	17.38 (11.67)		8.48 (6.56)		37.49 (8.69)	
Type of employment contract						
Permanent	17.28 (11.37)		8.20 (6.40)		37.44 (8.57)	
Indefinite	17.85 (11.31)	0.54	9.61 (6.06)	0.52	38.18 (7.98)	0.57
Part-time	14.65 (10.71)		8.22 (6.49)		39.43 (8.18)	
**Variables**	**EE**	***p*-Value**	**DP**	***p*-Value**	**PA**	***p*-Value**
	**CC**		**CC**		**CC**	
Age (years)	0.09	0.25	-0.06	0.41	-0.10	0.03
Time of service at the ED (years)	0.07	0.37	0.01	0.87	-0.17	0.03
Work experience (years)	0.10	0.20	-0.07	0.35	-0.08	0.02

M: Mean; SD: Standard deviation; EE: Emotional exhaustion; DP: Depersonalization; PA: Personal accomplishment; ED: Emergency department; CC: Correlation coefficient.

**Table 4:** Multivariate analysis for the variables predicting each dimension of burnout.

9	EE *				DP **				PA ***			
	B	*p*-Value	*ß*	*p*-Value	B	*p*-Value	*ß*	*p*-Value	B	*p*-Value	*ß*	*p*-Value
Sex												
Male	Ref	0.02	Ref	0.01	Ref	0.007	Ref	0.01	Ref	0.40		
Female	4.59		4.59		2.92		2.74		−1.23			
Marital status												
Married	Ref	0.80			Ref	0.29			Ref	0.77		
Not married	0.45				−1.06				−0.40			
Daily physical exercise												
Yes	Ref	0.09			Ref	0.005	Ref	0.006	Ref	0.48		
No	3.51				2.72		2.47		−0.92			
Daily tobacco use												
Yes	Ref	0.69			Ref	0.99			Ref	0.58		
No	0.68				0.02				−0.72			
Type of employment contract												
	Ref	0.69			Ref	0.42			Ref	0.36		
	−0.74				0.84				1.25			
Age (years)	0.12	0.25			−0.05	0.41			−0.10	0.02		
Time of service at the ED (years)	0.08	0.37			0.008	0.87			−0.14	0.03	−0.14	0.03
Work experience (years)	0.13	0.20			−0.05	0.35			−0.08	0.03		
Perceived stress (points)	0.01	0.93			0.13	0.10			−0.07	0.54		
Job satisfaction (points)	−0.07	0.57			−0.003	0.97			−0.08	0.41		
Somatic symptoms (points)	0.44	0.06			0.01	0.91			−0.08	0.64		
Anxiety (points)	0.23	0.02	0.11	0.01	−0.005	0.97			−0.12	0.45		
Social dysfunction (points)	0.53	0.03	0.39	0.02	−0.10	0.57			−0.18	0.42		
Depression (points)	0.26	0.12			0.02	0.86			0.11	0.35		
Problem-focused coping (points)	−2.30	0.17			−2.03	0.18			1.07	0.40		
Emotion-focused coping (points)	1.17	0.62			0.46	0.73			0.21	0.91		
Avoidance coping (points)	3.31	0.02	3.30	0.04	1.34	0.02	1.02	0.03	0.64	0.66		

B = Estimated parameter; Ref: Reference; EE: Emotional exhaustion; DP: Depersonalization; PA: Personal accomplishment. * Adjusted R-Squared for emotional exhaustion = 0.04, *F* = 4.46, *p* = 0.01; ** Adjusted R-Squared for depersonalization = 0.07, *F* = 7.63, *p* = 0.0007; *** Adjusted R-Squared for personal accomplishment = 0.02, *F* = 4.89, *p* = 0.03.

## Supplementary Materials

The following are available online at https://www.mdpi.com/2077-0383/9/4/1007/s1, File S1. Research data.
